# Changes of serum pentraxin-3 and hypersensitive CRP levels during pregnancy and their relationship with gestational diabetes mellitus

**DOI:** 10.1371/journal.pone.0224739

**Published:** 2019-11-13

**Authors:** Ning Yu, Hongyan Cui, Xu Chen, Ying Chang

**Affiliations:** Department of Obstetrics, Tianjin Central Hospital of Obstetrics and Gynecology, Tianjin Key Laboratory of Human Development and Reproductive Regulation, Tianjin, China; Eunice Kennefy Shriver National Institute of Child Health and Human Development (NICHD), National Institutes of Health (NIH), UNITED STATES

## Abstract

**Objective:**

To investigate the changes of inflammatory factors pentraxin 3 (PTX3) and hypersensitive C-reactive protein (hs-CRP) during pregnancy and their relationship with gestational diabetes mellitus (GDM).

**Methods:**

The nested case-control study method was used. Eighty non-obese single-pregnant women diagnosed with GDM were included into the case group (GDM, n = 80), together with another eighty pregnant women with normal glucose tolerance were matched in the same period and divided into the control group (CON, n = 80), for detecting multiple biochemical indicators in different pregnancy stages by ELISA.

**Results:**

The serum levels of PTX3 and hs-CRP in pregnant women increased with the increase of gestational age (p < 0.001, p < 0.001). The levels of PTX3 and hs-CRP in group GDM were significantly higher in the middle and late pregnancy stages than group CON (p < 0.01, p < 0.05; p < 0.05, p < 0.05). PTX3 was positively correlated with hs-CRP, body mass index (BMI), fasting plasma glucose (FPG), and homeostasis model assessment of insulin resistance (HOMAIR).

**Conclusions:**

PTX3 and hs-CRP may be related to the pathogenesis of GDM, and they are significantly increased in the second trimester, which provides a new idea for early prevention and treatment of GDM and risk prediction of long-term cardiovascular diseases.

## Introduction

GDM refers to abnormal glucose metabolism that occurs or is discovered during pregnancy, it can cause multiple poor outcomes and increase maternal and child prevalence and mortality. In addition, GDM can increase the risk of type 2 diabetes mellitus (T2DM) and cardiovascular diseases in pregnant women, and the risk of metabolic disorders in their offspring is high, so it’s called a disease that affects two generations [1]. Studies have shown that GDM is an important and variable risk factor for T2DM and prevention of GDM can reduce the incidence of T2DM in offspring [2]. It has been found that there is an acute phase inflammatory response in women with hyperglycemia during pregnancy, and this low inflammatory state of the body is considered to be related to the pathogenesis of GDM.

PTX3 and CRP are both the acute phase proteins belonging to the pentraxin family but with different biological characteristics, CRP belongs to the short PTX, which is an acute phase protein synthesized by the hepatocytes when the body is stimulated by microbial invasion or tissue damage [3], and increases in the early stages of tissue damage [4–6]. PTX3 is a long-chain PTX protein. A variety of tissue cells can produce PTX3 under the stimulation of pro-inflammatory factors, including the endothelial cells, fibroblasts, monocytes, adipocytes, etc [7,8]. so PTX3 and hs-CRP can be combined to study the relevance of diseases.

In recent years, research on PTX3/hs-CRP and metabolic syndrome, cardiovascular disease, and DM/DM-related complications has been increasing, but the results of various studies are inconsistent, and some are even contradictory. At the same time, it is rarely reported on PTX3/hs-CRP and GDM patients, a special population with high risk of secondary metabolic disorders, and whether hs-CRP and PTX3 have synergy in the occurrence of diseases is still unclear. This article investigated the changes of PTX3 and hs-CRP levels in pregnant women by detecting serum PTX3, hs-CRP, and related biochemical indicators in pregnant women at different stages (early, middle and late), and analyzed the correlation between PTX3/hs-CRP and GDM, aiming to explore the early predictive value of inflammatory markers (PTX3 and hs-CRP) for the pathogenesis of GDM.

## Materials and methods

### Objects

The nested case-control study method was used. The single-fetal primiparas in the early pregnancy (11–14 weeks) were tested in the outpatient department of Tianjin Central Hospital of Obstetrics and Gynecology (between January 2016 and January 2017) and excluded from obesity, fetal malformation, or related medical problems and family history. The blood samples were collected during the early pregnancy. Of 600 pregnancies enrolled, 10 were lost to follow-up and 12 underwent spontaneous abortion. 578 cases were performed 75 g oral glucose tolerance test (OGTT) in the second trimester (24–28 weeks). The non-obese patients diagnosed with GDM were divided into group GDM (n = 80), and non-obese pregnant women with normal glucose tolerance matched with the same age and gestational age were selected as group CON (n = 80). The blood samples were collected during the second trimester of pregnancy. The serum PTX3 and hs-CRP levels were measured in the early and middle trimesters of the two groups. All the cases were followed up until delivery, and blood samples were collected before delivery in the third trimester of pregnancy. Obese cases were excluded by BMI ≥ 30, and the biochemical indicators such as serum PTX3, hs-CRP, FPG, fasting insulin (FINS), triglyceride (TG), and total cholesterol (TCH) were tested. This study was conducted in accordance with the declaration of Helsinki. This study was conducted with approval from the Ethics Committee of Tianjin Central Hospital of Obstetrics and Gynecology. Written informed consent was obtained from all participants.

### Diagnostic criteria

We adopted the diagnostic criteria of the American Diabetes Association (ADA) [9]: blood glucose levels should be less than 5.1, 10, and 8.5 mmol / L before, 1, and 2 hr after taking sugar. Any of the above blood glucose level that met or exceeded the above criteria was diagnosed as GDM.

### Determination of PTX3/hs-CRP and biochemical indicators

3 ml of blood was collected from the antecubetal fossa and centrifuged at 2683 g for 10 minutes to obtain the serum, which was stored -20°C. The serum samples of group GDM and group CON were obtained from stored blood and performed freezing/thawing once.

Serum PTX3 concentration was detected by specific and sensitive enzyme-linked immunoassays (ELISA) (the kit was purchased from Boster Bioengineering Co., Ltd. USA, catalog No. EK0861); serum hs-CRP was detected by ELISA (the kit was purchased from Wuhan Huamei Bioengineering Co., Ltd. CUSABIO, China, catalog No. CSB-E08617h); FPG, TG, and TCH were measured using one automatic biochemical analyzer; FINS was determined by chemiluminescence.

### Statistical analysis

The insulin resistance (IR) condition was evaluated using the HOMA model, HOMAIR has been proved to be a robust tool for the assessment of IR and the index of IR that is most widely used in studies [10]. HOMAIR = FINS x FPG/22.5. The Kolmogorov-Smirnov method was used to verify whether the data were in normal distribution. The measurement data were expressed as $\bar{x}\pm \text{s}$. The mean comparison between two groups was performed by the t test. The mean comparison among groups was analyzed by one-way ANOVA, and the comparison between two indices was performed by the SNK q test. The Pearson correlation analysis was used for correlation analysis among variables. Statistical analysis was performed using SPSS 19.0, with *P* < 0.05 being considered as statistical significance.

## Results

### Comparison of general clinical data

There were nosignificant differences in the general data of age, maternal birth, early pregnancy BMI, and mean blood sampling gestational age between the two groups in three different stages of pregnancy (early 11–14 weeks, mid-term 24–28 weeks, late 36–40 weeks) (p > 0.05) (Table 1).

**Table 1:** Comparison of clinical data between two groups ($\bar{x}\pm \text{s}$).

	GDM (n = 80)	CON (n = 80)	p
Age (years)	28.6±4.2	26.9±3.6	0.321
BMI (Kg/m^2^)	24.2±3.5	23.5±4.7	0.428

### Changes of serum PTX3 and hs-CRP levels in different stages of pregnancy

**Fig 1:**
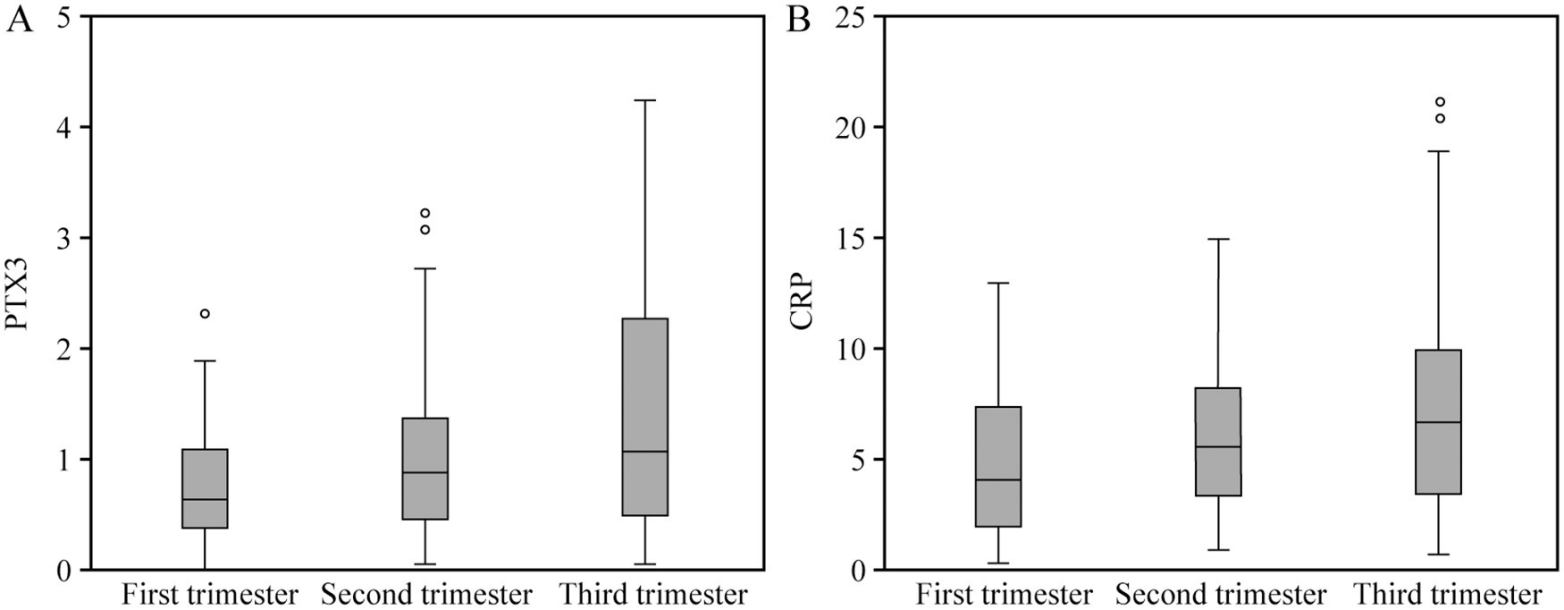
Comparison of serum PTX3 and hs-CRP llevels in different periods of pregnancy.

In the late pregnancy, 21 cases in group GDM and 12 cases in group CON were excluded due to BMI ≥ 30, so the number of cases in the third trimester was 127 cases. The serum levels of PTX3 and hs-CRP in pregnant women increased with the increase of gestational age (p < 0.001, p < 0.001). The serum PTX3 level in the early pregnancy was significantly lower than those in the middle and late pregnancy (p = 0.022, p < 0.001), and the serum PTX3 level in the second trimester was significantly lower than that in the third trimester (p = 0.048). The serum hs-CRP level in the early pregnancy was significantly lower than those in the middle and late pregnancy (p = 0.031, p < 0.001), and the serum hs-CRP level in the second trimester was significantly lower than that in the third trimester (p = 0.049) (Fig 1).

### Comparison of serum PTX3 and hs-CRP levels between GDM and CON at different stages of pregnancy

**Fig 2:**
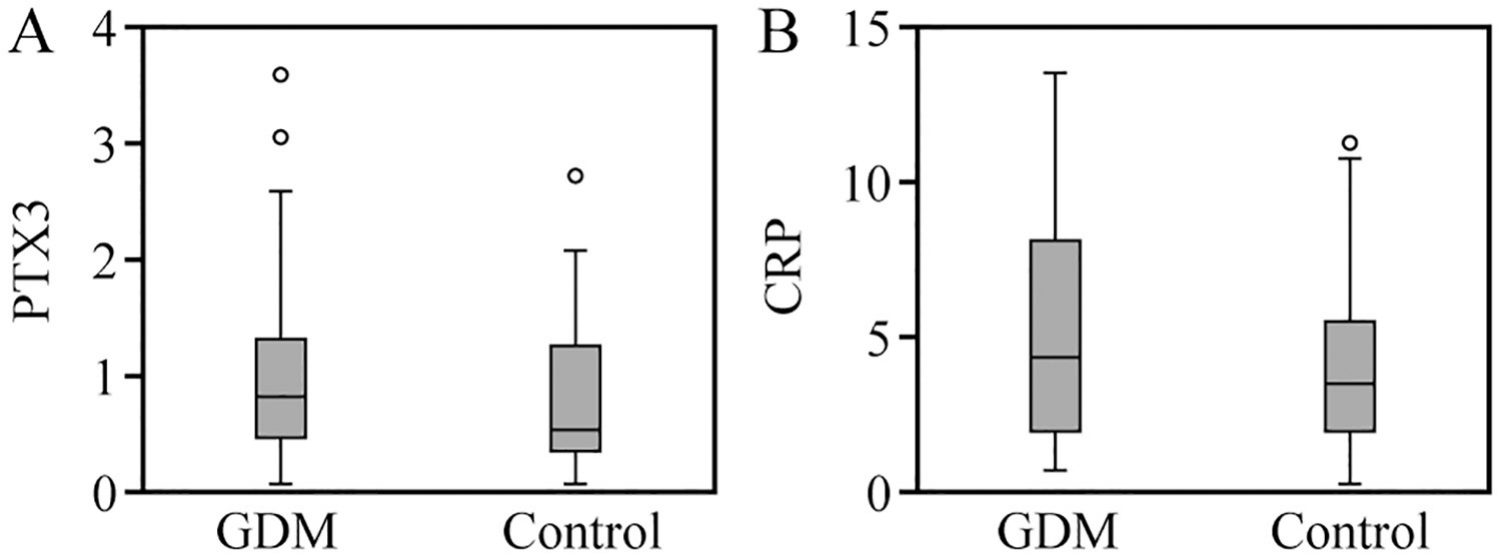
Comparison of serum PTX3 and hs-CRP levels between two groups in early pregnancy.

**Fig 3:**
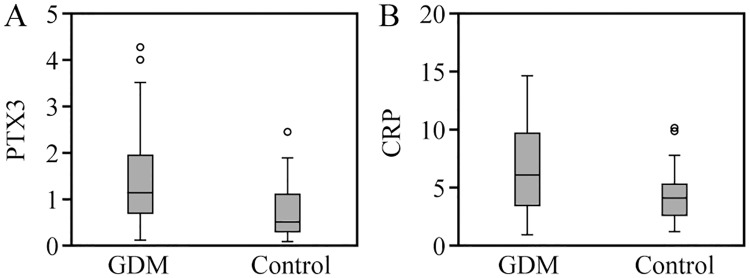
Comparison of serum PTX3 and hs-CRP levels between two groups in middle pregnancy.

**Fig 4:**
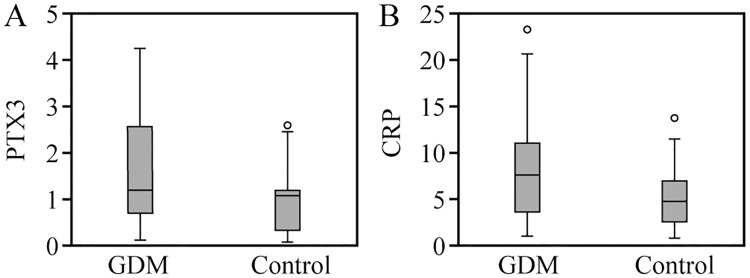
Comparison of serum PTX3 and hs-CRP levels between two groups in late pregnancy.

The levels of serum PTX3 and hs-CRP in the two groups increased with the increase of gestational age. There was no significant difference in the serum PTX3 and hs-CRP levels between the two groups in the early pregnancy (p > 0.05, p > 0.05). The serum levels of PTX3 and hs-CRP in group GDM were significantly higher than group CON in the middle pregnancy (p < 0.01, p < 0.05), as shown in Figs 2 and 3. In the late pregnancy, 21 patients in group GDM and 12 patients in group CON were excluded due to BMI ≥ 30. In the third trimester, the serum PTX3 and hs-CRP levels in group GDM (59 patients) were significantly higher than those in group CON (68 patients), (p < 0.05, p < 0.05) (Fig 4).

### Comparison of biochemical indicators in late pregnancy

There was no significant difference in BMI between group GDM and group CON (p > 0.05). The levels of FPG and HOMAIR were significantly higher in group GDM than group CON (p < 0.001, p < 0.001), but there were no significant differences in serum lipids (TG and TCH) between the two groups (p > 0.05, p > 0.05) (Table 2).

**Table 2:** Comparison of biochemical indicators between two groups in late pregnancy ($\bar{x}\pm \text{s}$).

	GDM (n = 59)	CON (n = 68)	p
FPG (mmol/L)	*5*.*81±2*.*45*	4.29±0.86	0.000
HOMA_IR_	3.92±1.58	2.03±0.62	0.000
TG (mmol/L)	2.94±0.83	2.05±0.51	0.332
TCH (mmol/L)	6.86±1.92	5.92±1.63	0.570
BMI (Kg/m^2^)	27.68±1.45	25.47±2.48	0.886

### Correlation analysis

PTX3 was positively correlated with hs-CRP, BMI, FPG, and HOMAIR (r = 0.532, 0.584, 0.677, 0.718, p = 0.016, 0.028, 0.011, 0.009), and hs-CRP was positively correlated with BMI, PTX3, FPG, and HOMAIR (r). = 0.472, 0.532, 0.637, 0.763, p = 0.036, 0.028, 0.012, 0.005). There was no correlation between PTX3/hs-CRP and blood lipids (TG, TCH) (r = 0.017, p = 0.872; r = 0.042, p = 0.903).

## Discussion

The main purpose of this paper was to investigate the association between inflammatory factor PTX3/hs-CRP and the pathogenesis of GDM, and whether they can work synergistically. Our results showed that the serum PTX3 and hs-CRP levels in GDM and normal pregnant women increased with gestational age (11–14 weeks, 24–28 weeks, 36–40 weeks). There were no significant differences in the serum PTX3 and hs-CRP levels between the two groups in early pregnancy. The levels of serum PTX3 and hs-CRP in group GDM were higher than group CON from the middle of pregnancy. The levels of inflammatory factors in group GDM were also significantly higher than group CON in the third trimester. The correlation analysis showed that both PTX3 and hs-CRP were positively correlated with BMI, FPG, and HOMAIR while not related to blood lipids. At the same time, these two were positively correlated with each other. Inflammatory factors such as interleukin type 6 (IL-6) and tumor necrosis factor-alpha (TNF-α)] result in increased gestational infection and stimulation of acute phase inflammatory response [11]. PTX3 and CRP are both the acute phase inflammatory proteins and participate in the first phase of the immune response [12]. Studies have also shown the association between GDM and inflammatory factors and stress as well as oxidation [13]. Therefore, the two should play important roles on inducting GDM. These results suggest that PTX3 and hs-CRP are associated with abnormal glucose metabolism, are associated with the pathogenesis of GDM, and may work together, consistent with our hypothesis.

During pregnancy, the body’s immune system and endocrine system will undergo a series of physiological changes. Normal pregnancy itself has physiological IR, which is an adaptive change in vivo. As pregnancy progresses, pregnant women’s blood is diluted, so the insulin secretion is relatively insufficient while the hormones secreted by the placenta to antagonize insulin gradually increase, thus leading to a gradual increase in the degree of IR. If the insulin secreted by the body can’t be compensated, GDM occurs. At present, there are few reports on the changes of PTX3 level changes during pregnancy. Lekva et al [14] once suggested that the serum PTX3 levels in pregnant women are on the rise, consistent with our findings; the trend of increase in GDM patients is more pronounced than normal pregnant women. These results indicate that the level of inflammatory factors in the body increases with the advance of physiological IR during pregnancy, suggesting that the inflammatory state is related to the occurrence and development of IR, which may be related to the pathogenesis of GDM.

In recent years, many studies have shown that metabolic syndrome is associated with inflammatory mediators, the most representative of which is hs-CRP: elevated serum hs-CRP level has been found in patients with diabetes, hypertension, obesity, hyperlipidemia, and even cardiovascular diseases [15]. PTX3 is similar to CRP in structure and function and has a good correlation with hs-CRP. It also supports the low-inflammation background of patients with metabolic syndrome, and is also an independent predictor of cardiovascular disease as CRP [16]. GDM is the main manifestation of metabolic disorders during pregnancy. Our study found that serum PTX3 and hs-CRP in GDM pregnant women were higher than normal pregnant women, and they were positively correlated, indicating that these two inflammatory factors are related to abnormal glucose metabolism during pregnancy, and jointly promote this a process. Because obesity is also one of the metabolic diseases, in this study, we excluded obese pregnant women by BMI to reduce the associated confounding factors.

The pathogenesis of GDM is still unclear, but as a "pioneer" of T2DM, it seems to be related to inflammatory pathways and endocrine metabolic mechanism, even there are studies revealing that GDM pregnant women have inflammatory disorders in early pregnancy [14]. Yildirim et al [17] prospectively investigated the correlation between PTX3 and GDM, and the results showed that the levels of PTX3 were significantly higher than normal pregnant women, and the PTX3 was positively correlated with the 50 g challenge test, so it’s considered that the maternal serum PTX3 level is associated with hyperglycemia, which may be the vascular pathological indicator of GDM during the OGTT period. Todoric et al [18] studied the role of PTX3 in GDM, detected the fasting serum PTX3 level of 90 pregnant women, as well as the serum PTX3 level in 20 pregnant women with 24–28 weeks 2 hours after OGTT test. The level of PTX3 in group GDM was significantly higher than group CON, and the increase of PTX3 concentration in group GDM was significantly higher than group CON2 hours after taking sugar, suggesting that the blood glucose is positively correlated with PTX3 while negatively correlated with insulin sensitivity. Our study also found that PTX3 was positively correlated with FPG and IR, consistent with the above findings, and this inflammatory factor appeared differently in the second trimester, indicating its predictive value for GDM risk. However, there are also different studies suggesting that PTX3 levels are negatively correlated with IR, suggesting a potential protective function of PTX3 [14]. The reasons for the different conclusions of the existing research are unclear and may be related to the sample size or the diagnostic criteria of the study objects.

For the relationship between hs-CRP and GDM, current studies have shown that the hs-CRP level in the first and second trimesters is positively correlated with the risk of GDM, as well as positively correlated with FPG, obesity, and IR; meanwhile, the hs-CRP level in the second trimester is also related to the adverse pregnancy outcomes [19–21]. Our study also found that the serum hs-CRP level in GDM pregnant women was significantly higher and positively correlated with IR and FPG, and this change began to appear in the second trimester and further aggravated in the third trimester. It can be seen that the chronic inflammatory reaction is accompanied by the development of GDM, and becomes more obvious as the pregnancy progresses and the abnormal blood glucose aggravates, suggesting that inflammatory factors may not be negligible during the occurrence and development of GDM and has early predictive value.

The results of this paper also have some limitations. First, the data came from a single center, and the sample size was limited. The correlation among PTX3, hs-CRP, and GDM needs more multi-center, large-sample research data for confirmation, and we will continue to deepen such studies. Second, there are still many problems to be solved in the practical application of PTX3 and hs-CRP, such as the sensitivity and specificity of the two factors, at what level, and what clinical intervention is needed; we will keep on investigating these in follow-up studies. Third, although clinical infections have been excluded, we still can’t guarantee that there will be no asymptomatic infections that may affect the PTX3 and hs-CRP levels. The levels of PTX3 and hs-CRP are significantly higher in GDM women from the second trimester, and positively correlated with FPG and IR, indicating that when abnormal glucose metabolism begins, a status of low-degree inflammation also occurs in the body, which further confirms the relationship of inflammation and incidence of GDM. Findings showed a higher BMI and HOMAIR in women with GDM compared with healthy pregnant women [22, 23], but many of them are related to obesity. This study excluded obesity before pregnancy and related family history. So, PTX3 combined with hs-CRP can be used as early screening indicators for GDM to facilitate early diagnosis and treatment of GDM, especially in low-risk pregnant women. The time of PTX3 release into peripheral blood (6–8 h) is much faster than CRP (24 h), and it can exist in peripheral blood and tissues for a long time, of which hs-CRP is unmatchable. Therefore, monitoring the serum PTX3 level in patients with GDM may be more direct and more effective, but the sensitivity may be slightly worse than hs-CRP. In addition, subclinical inflammation is also a major risk factor for cardiovascular diseases, and pregnant women with a history of GDM have an increased risk of developing cardiovascular diseases in the future, at least in partial reasons. Therefore, the emergence of inflammatory factors PTX3 and hs-CRP also provide new ideas for the early prevention and treatment of GDM and the prediction of long-term cardiovascular disease risk.

## Supporting information

S1 File PLOSOne_Clinical_Studies_Checklist. (DOCX)

S2 File STROBE_checklist_v4_combined_PlosMedicine. (DOCX)

## Abbreviations

ADA: American Diabetes Association
BMI: body mass index
CON: control group
ELISA: enzyme-linked immunoassays
FINS: fasting insulin
GDM: gestational diabetes mellitus
HOMAIR: homeostasis model assessment of insulin resistance
hs-CRP: hypersensitive C-reactive protein
IL-6: interleukin type 6
IR: insulin resistance
OGTT: oral glucose tolerance test
PTX3: pentraxin 3
T2DM: type 2 diabetes mellitus
TCH: total cholesterol
TG: triglyceride
TNF-α: tumor necrosis factor-alpha
