# Supplementary material for: Changes of serum pentraxin-3 and hypersensitive CRP levels during pregnancy and their relationship with gestational diabetes mellitus
Source: PLoS One. 2019 Nov 13;14(11):e0224739. doi: 10.1371/journal.pone.0224739 (PMC6853302; doi:10.1371/journal.pone.0224739)
Supplement: S2 File — (DOCX) [file pone.0224739.s002.docx]

STROBE Statement—checklist of items that should be included in reports of observational studies

|  | Item No. | Recommendation | Page  No. | Relevant text from manuscript |
| --- | --- | --- | --- | --- |
| **Title and abstract** | 1 | (*a*) Indicate the study’s design with a commonly used term in the title or the abstract | 2 | The nested case-control study method was used. |
|  |  | (*b*) Provide in the abstract an informative and balanced summary of what was done and what was found | 2 | PTX3 and hs-CRP may be related to the pathogenesis of GDM, and they are significantly increased in the second trimester, which provides a newidea for early prevention and treatment of GDM and risk prediction of long-term cardiovascular diseases. |
| Introduction | | | |  |
| Background/rationale | 2 | Explain the scientific background and rationale for the investigation being reported | 3 | It has been found that there is an acute phase inflammatory response in women with hyperglycemia during pregnancy, and this low inflammatory state of the body is considered to be related to the pathogenesis of GDM. |
| Objectives | 3 | State specific objectives, including any prespecified hypotheses | 4 | This article investigated the changes of PTX3 and hs-CRP levels in pregnant women by detecting serum PTX3, hs-CRP, and related biochemical indicators in pregnant women at different stages (early, middle and late), and analyzed the correlation between PTX3/hs-CRP and GDM, aiming to explore the early predictive value of inflammatory markers (PTX3 and hs-CRP) for the pathogenesis of GDM. |
| Methods | | | |  |
| Study design | 4 | Present key elements of study design early in the paper | 4 | The nested case-control study method was used. |
| Setting | 5 | Describe the setting, locations, and relevant dates, including periods of recruitment, exposure, follow-up, and data collection | 4 | The single-fetal primiparas in the early pregnancy (11-14 weeks) were tested in the outpatient department of Tianjin Central Hospital of Obstetrics and Gynecology (between January 2016 and January 2017) |
| Participants | 6 | (*a*) *Cohort study*—Give the eligibility criteria, and the sources and methods of selection of participants. Describe methods of follow-up  *Case-control study*—Give the eligibility criteria, and the sources and methods of case ascertainment and control selection. Give the rationale for the choice of cases and controls  *Cross-sectional study*—Give the eligibility criteria, and the sources and methods of selection of participants | 4, 5 | The single-fetal primiparas in the early pregnancy (11-14 weeks) were tested in the outpatient department of Tianjin Central Hospital of Obstetrics and Gynecology (between January 2016 and January 2017) and excluded from obesity, fetal malformation, or related medical problems. The blood samples were collected during the early pregnancy. The selected cases were performed 75 g OGTT in the second trimester (24-28 weeks). The non-obese patients diagnosed with GDM were divided into group GDM, and non-obese pregnant women with normal glucose tolerance matched with the same age and gestational age were selected as group CON. |
|  |  | (*b*)*Cohort study*—For matched studies, give matching criteria and number of exposed and unexposed  *Case-control study*—For matched studies, give matching criteria and the number of controls per case | 5 | The non-obese patients diagnosed with GDM were divided into group GDM, and non-obese pregnant women with normal glucose tolerance matched with the same age and gestational age were selected as group CON. |
| Variables | 7 | Clearly define all outcomes, exposures, predictors, potential confounders, and effect modifiers. Give diagnostic criteria, if applicable | 5 | Obese cases were excluded by BMI ≥ 30. Diagnostic criteria of the American Diabetes Association (ADA): blood glucose levels should be less than 5.1, 10, and 8.5 mmol / L before, 1, and 2 hr after taking sugar. Any of the above blood glucose level that meets or exceeds the above criteria is diagnosed as GDM. |
| Data sources/measurement | 8* | For each variable of interest, give sources of data and details of methods of assessment (measurement). Describe comparability of assessment methods if there is more than one group | 6 | Serum PTX3 concentration was detected by specific and sensitive enzyme-linked immunoassays (ELISA) (the kit was purchased from Boster Bioengineering Co., Ltd. USA); serum hs-CRP was detected by ELISA (the kit was purchased from Wuhan Huamei Bioengineering Co., Ltd. CUSABIO, China); FPG, TG, and TCH were measured using one automatic biochemical analyzer; FINS was determined by chemiluminescence. |
| Bias | 9 | Describe any efforts to address potential sources of bias | 4, 6 | Excluded from obesity, fetal malformation, or related medical problems. 3 ml of blood was collected from the elbow vein and centrifuged at 4000 r/min for 10 minutes to obtain the serum, which was stored -20°C. The serum samples of group GDM and group CON were obtained from stored blood and performed freezing/thawing once. |
| Study size | 10 | Explain how the study size was arrived at | 4, 5 | The single-fetal primiparas in the early pregnancy (11-14 weeks) were tested in the outpatient department of Tianjin Central Hospital of Obstetrics and Gynecology (between January 2016 and January 2017) and excluded from obesity, fetal malformation, or related medical problems. All the cases were followed up until delivery |

Continued on next page

| Quantitative variables | 11 | Explain how quantitative variables were handled in the analyses. If applicable, describe which groupings were chosen and why | 6 | The Kolmogorov-Smirnov method was used to verify whether the data were in normal distribution. The measurement data were expressed as $\overline{x}$±s. |
| --- | --- | --- | --- | --- |
| Statistical methods | 12 | (*a*) Describe all statistical methods, including those used to control for confounding | 6 | The mean comparison between two groups was performed by the t test. The mean comparison among groups was analyzed by one-way ANOVA, and the comparison between two indexes was performed by the SNK q test. The Pearson correlation analysis was used for correlation analysis among variables. Statistical analysis was performed using SPSS 19.0, with *P* < 0.05 being considered as statistical significance. |
|  |  | (*b*) Describe any methods used to examine subgroups and interactions | 6 | the comparison between two indexes was performed by the SNK q test. |
|  |  | (*c*) Explain how missing data were addressed | 6 | excluding |
|  |  | (*d*) *Cohort study*—If applicable, explain how loss to follow-up was addressed  *Case-control study*—If applicable, explain how matching of cases and controls was addressed  *Cross-sectional study*—If applicable, describe analytical methods taking account of sampling strategy | 5 | matched with the same age and gestational age |
|  |  | (*e*) Describe any sensitivity analyses | - | - |
| Results | | | | |
| Participants | 13* | (a) Report numbers of individuals at each stage of study—eg numbers potentially eligible, examined for eligibility, confirmed eligible, included in the study, completing follow-up, and analysed | 19, 20 | Table 2, 3,4 |
|  |  | (b) Give reasons for non-participation at each stage | 8 | In the late pregnancy, 21 patients in group GDM and 12 patients in group CON were excluded due to BMI ≥ 30. In the third trimester, the serum PTX3 and hs-CRP levels in group GDM (59 patients) were significantly higher than those in group CON (68 patients), (P < 0.05, P < 0.05) |
|  |  | (c) Consider use of a flow diagram | - | - |
| Descriptive data | 14* | (a) Give characteristics of study participants (eg demographic, clinical, social) and information on exposures and potential confounders | 7 | There were nosignificant differences in the general data of age, maternal birth, early pregnancy BMI, and mean blood sampling gestational age between the two groups in three different stages of pregnancy (early 11-14 weeks, mid-term 24-28 weeks, late 36-40 weeks) (*P* > 0.05) (Table 1). |
|  |  | (b) Indicate number of participants with missing data for each variable of interest | - | - |
|  |  | (c) *Cohort study*—Summarise follow-up time (eg, average and total amount) | - | - |
| Outcome data | 15* | *Cohort study*—Report numbers of outcome events or summary measures over time | *-* | *-* |
|  |  | *Case-control study—*Report numbers in each exposure category, or summary measures of exposure | 19, 20 | Table 2, 3,4 |
|  |  | *Cross-sectional study—*Report numbers of outcome events or summary measures | *-* | *-* |
| Main results | 16 | (*a*) Give unadjusted estimates and, if applicable, confounder-adjusted estimates and their precision (eg, 95% confidence interval). Make clear which confounders were adjusted for and why they were included | 19, 20 | Table 2, 3, 4, 5 |
|  |  | (*b*) Report category boundaries when continuous variables were categorized | - | - |
|  |  | (*c*) If relevant, consider translating estimates of relative risk into absolute risk for a meaningful time period | 8 | PTX3 was positively correlated with hs-CRP, BMI, FPG, and HOMAIR (r = 0.532, 0.584, 0.677, 0.718, P = 0.016, 0.028, 0.011, 0.009), and hs-CRP was positively correlated with BMI, PTX3, FPG, and HOMAIR (r). = 0.472, 0.532, 0.637, 0.763, P = 0.036, 0.028, 0.012, 0.005). There was no correlation between PTX3\hs-CRP and blood lipids (TG, TCH) (r = 0.017, P = 0.872; r = 0.042, P = 0.903). |

Continued on next page

| Other analyses | 17 | Report other analyses done—eg analyses of subgroups and interactions, and sensitivity analyses | - | - |
| --- | --- | --- | --- | --- |
| Discussion | | | | |
| Key results | 18 | Summarise key results with reference to study objectives | 9 | Our results showed that the serum PTX3 and hs-CRP levels in GDM and normal pregnant women increased with gestational age (11-14 weeks, 24-28 weeks, 36-40 weeks). There were no significant differences in the serum PTX3 and hs-CRP levels between the two groups in early pregnancy. The levels of serum PTX3 and hs-CRP in group GDM were higher than group CON from the middle of pregnancy. The levels of inflammatory factors in group GDM were also significantly higher than group CON in the third trimester. The correlation analysis showed that both PTX3 and hs-CRP were positively correlated with BMI, FPG, and HOMAIR while not related to blood lipids. At the same time, these two were positively correlated with each other. These results suggest that PTX3 and hs-CRP are associated with abnormal glucose metabolism, are associated with the pathogenesis of GDM, and may work together, consistent with our hypothesis. |
| Limitations | 19 | Discuss limitations of the study, taking into account sources of potential bias or imprecision. Discuss both direction and magnitude of any potential bias | 13, 14 | The results of this paper also have some limitations. First, the data came from a single center, and the sample size was limited. The correlation among PTX3, hs-CRP, and GDM needs more multi-center, large-sample research data for confirmation, and we will continue to deepen such studies. Second, there are still many problems to be solved in the practical application of PTX3 and hs-CRP, such as the sensitivity and specificity of the two factors, at what level, and what clinical intervention is needed; we will keep on investigating these in follow-up studies. Third, although clinical infections have been excluded, we still can’t guarantee that there will be no asymptomatic infections that may affect the PTX3 and hs-CRP levels. |
| Interpretation | 20 | Give a cautious overall interpretation of results considering objectives, limitations, multiplicity of analyses, results from similar studies, and other relevant evidence | 10-12 | Lekva et al [10] once suggested that the serum PTX3 levels in GDM and non-GDM pregnant women are on the rise (14-16 weeks, 22-24 weeks, 36-38 weeks). Karakas et al [13] studied the correlation between PTX3 and metabolic syndrome and found that the level of PTX3 in the blood circulation of patients with metabolic syndrome is significantly higher than that in normal populations, and serum PTX3 and hs-CRP levels are related to the severity of the disease, increasing with the degree of metabolic disorders. Zanetti et al [14] also reported that serum PTX3 and hs-CRP levels are significantly elevated in patients with metabolic syndrome and may be associated with inflammation and subclinical atherosclerosis. Todoric et al [17] studied the role of PTX3 in GDM, The level of PTX3 in group GDM was significantly higher than group CON, and the increase of PTX3 concentration in group GDM was significantly higher than group NGT 2 hours after taking sugar, suggesting that the blood glucose is positively correlated with PTX3 while negatively correlated with insulin sensitivity. |
| Generalisability | 21 | Discuss the generalisability (external validity) of the study results | 14, 15 | he levels of PTX3 and hs-CRP in pregnant women are significantly higher than those in normal pregnant women from the second trimester, and positively correlated with FPG and IR, indicating that when abnormal glucose metabolism begins, a status of low-degree inflammation also occurs in the body, which further confirms the relationship of inflammation and incidence of GDM. So, PTX3 combined with hs-CRP can be used as early screening indicators for GDM to facilitate early diagnosis and treatment of GDM. In addition, subclinical inflammation is also a major risk factor for cardiovascular diseases, and pregnant women with a history of GDM have an increased risk of developing cardiovascular diseases in the future, at least in partial reasons. Therefore, the emergence of inflammatory factors PTX3 and hs-CRP also provide new ideas for the early prevention and treatment of GDM and the prediction of long-term cardiovascular disease risk. |
| Other information | |  | | |
| Funding | 22 | Give the source of funding and the role of the funders for the present study and, if applicable, for the original study on which the present article is based | 15 | This work was funded by Tianjin municipal health and family planning commission (2015KZ079). |

*Give information separately for cases and controls in case-control studies and, if applicable, for exposed and unexposed groups in cohort and cross-sectional studies.

**Note:** An Explanation and Elaboration article discusses each checklist item and gives methodological background and published examples of transparent reporting. The STROBE checklist is best used in conjunction with this article (freely available on the Web sites of PLoS Medicine at http://www.plosmedicine.org/, Annals of Internal Medicine at http://www.annals.org/, and Epidemiology at http://www.epidem.com/). Information on the STROBE Initiative is available at www.strobe-statement.org.
